# The triglyceride and glucose index and risk of nonalcoholic fatty liver disease: A dose–response meta-analysis

**DOI:** 10.3389/fendo.2022.1043169

**Published:** 2023-01-19

**Authors:** Qin Ling, Jiawei Chen, Xiao Liu, Yi Xu, Jianyong Ma, Peng Yu, Kai Zheng, Fuwei Liu, Jun Luo

**Affiliations:** ^1^ Department of Cardiology, the Affiliated Ganzhou Hospital of Nanchang University, Jiangxi, China; ^2^ The Second Clinical Medical College of Nanchang University, Jiangxi, China; ^3^ Department of Cardiology, the Sun Yat-sen Memorial Hospital of Sun Yat-sen University, Guangzhou, China; ^4^ Department of Pharmacology and Systems Physiology, University of Cinnati College of Medicine, Cincinnati, OH, United States; ^5^ Department of Endocrine, the Second Affiliated Hospital of Nanchang University, Nanchang, Jiangxi, China; ^6^ Medical Care Strategic Customer Department, China Merchants Bank Shenzhen Branch, Shenzhen, China

**Keywords:** triglyceride and glucose index, nonalcoholic fatty liver disease, dose-response, meta-analysis, prognosis

## Abstract

**Background:**

The triglyceride and glucose (TyG) index is associated with the risk of nonalcoholic fatty liver disease (NAFLD), but the dose−response relationship between them is still unknown. We conducted a comprehensive meta-analysis to study the dose−response association between the TyG index and the risk of NAFLD.

**Methods:**

We systematically searched the Cochrane Library, PubMed, and Embase databases until July 2022 for relevant studies. The robust error meta-regression method was used to investigate the dose−response association between the TyG index and NAFLD. Summary relative risks (ORs) and 95% CIs were estimated by using a random-effects model.

**Results:**

A total of 4 cohort and 8 cross-sectional studies were included, with 28,788 NAFLD cases among the 105,365 participants. A positive association for the risk of NAFLD was observed for each additional unit of the TyG index with a linear association (p=0.82), and the summary OR was 2.84 (95% CI, 2.01-4.01). In the subgroup analyses, a stronger association of the TyG index with NAFLD was shown in females than in males (men: OR=2.97, 95% CI 2.55-3.46, women: OR=4.80, 95% CI 3.90-5.90, P_subgroup_<0.001).

**Conclusion:**

The TyG index may be a novel independent risk factor for NAFLD beyond traditional risk factors.

**Systematic Review Registration:**

https://www.crd.york.ac.uk/prospero, identifier (CRD42022347813).

## Introduction

Nonalcoholic fatty liver disease (NAFLD) is currently the most common chronic liver disease worldwide and not only leads to liver cirrhosis, liver failure or even liver cancer but also increases the risk of atherosclerosis and cardiovascular disease (CVD) (1). Therefore, early detection of patients at risk for NAFLD in a simple and effective manner is critical. However, the pathogenesis of NAFLD is still uncertain. The most popular pathomechanism is that insulin resistance (IR) plays a crucial role in the development of NAFLD (2). The triglyceride-glucose (TyG) index is calculated as LN (fasting triglyceride/fasting glucose) (3), which can usually be checked in healthy individuals. A recent study reported that the TyG index may be an alternative and reliable measure of IR. Studies have already pointed out that the TyG index is better in predicting the risk level of NAFLD patients compared with homeostasis model assessment-insulin resistance (4), a common diagnostic means for IR clinically. Zhang et al (5) and Zheng et al (6) published two studies in 2017 and 2018, respectively, both showing that the TyG index may be a predictor of incident NAFLD and concluding that it may be the best test for screening simple NAFLD.

Since then, multiple new studies regarding the relationship between TyG and NAFLD have been published, but the dose−response association between them is still unclear. Therefore, we aimed to evaluate the dose−response relationship between the TyG index and the risk of NAFLD in this study.

## Methods

### Protocol registration and search strategy

We have registered our study in the **International Prospective Register of Systematic Reviews** (PROSPERO) (https://www.crd.york.ac.uk/PROSPERO-CRD42022347813). As shown in **Supplemental Table 1**, we conducted this meta-analysis according to the Preferred Reporting Items for Systematic Reviews and Meta-Analyses (PRISMA) statement.

To find all studies on the relationship between the TyG index and NAFLD, we performed an exhaustive literature review through the PubMed and Embase databases and the Cochrane Library until July 18, 2022, and the concrete search strategy is provided in **Supplemental Table 2**. No date restriction was applied, but the English language and limitation to human studies were needed.

### Selection criteria and study selection

According to the population, intervention, comparison, outcome, and study design (known as the PICOS rules), the included criteria were as follows: (1) participants: adult (age>18 years); (2) comparison: high TyG index versus low TyG index; (3) outcomes: evaluated the relationship between the TyG index and the risk of NAFLD; (4) types of studies: observational studies published as full-length articles; and (5) reported the estimated effect for this association with multivariate analysis and provided useful data for the dose−response analysis. Therefore, studies were excluded for the following reasons: (1) they were reviews, meta-analyses, and congress abstracts; (2) they were abstract-only articles; (3) no relevant data were reported or could not be extracted; and (4) they used languages other than English.

Two investigators (Q-L and X-Y) separately completed the entire process of our study from the screening for included studies to data analysis. We identified the final included articles based on the title, abstract, and full text, and disagreements were resolved through coordination or by a third author (X-L) when necessary. An e-mail requesting the article or information was sent to the author when the article was not available or to obtain additional information for the analyses. Duplicated manuscripts were manually identified.

### Data collection and quality assessment

The following data were extracted by three independent researchers (Q-L, JW-C, Y-X) and examined for each eligible study by a fourth author (X-L): (1) name of first author; (2) year of publication; (3) country or region; (4) follow-up time; (5) baseline characteristics of the subjects (sample size, age, body mass index-BMI and so on); (6) study type; (7) outcome assessment; (8) number and percent of NAFLD incidence; (9) variables of adjustments; (10) TyG index value associated with the dose−response analysis; (11) hazard ratio (HR) or relative risk (RR) or odds ratio (OR) with 95% confidence interval (CI) from the most adjusted model.

The quality of the cross-sectional studies was judged using Joanna Briggs Institute’s critical appraisal checklist, while the cohort studies were evaluated using the Newcastle−Ottawa Scale (NOS). After an evaluation of selection, comparability, and outcomes, the studies were considered high-quality with an NOS of ≥6 stars.

### Statistical analysis

The majority of our included studies reported OR, and the others reported HR, and we uniformly downgraded HR to OR and merged the results. We used the one-stage method of robust error meta-regression (REMR) to fit the dose−response relationship between the TyG index and the risk of NAFLD (7, 8). In addition, we calculated the summary OR of the final results with the natural logarithm of the OR (log [OR]) and its standard error (SElog [OR]). The method of Greenland and Longnecker (9) was applied to compute study-specific slopes (linear trends) and 95% CIs.

Heterogeneity was assessed using the I^2^ statistics and Cochran Q test (p<0.1); I^2^>50% was regarded as high heterogeneity. To assess publication bias, a funnel plot, Egger’s test, and Begg’s test were performed. All analyses were performed using Stata 14.0 (Stata Corp LP, College Station, TX, USA) and Review Manager (RevMan) version 5.3 (The Cochrane Collaboration 2014; Nordic Cochrane Centre Copenhagen, Denmark).

## Results

### Literature search

**Figure 1:**
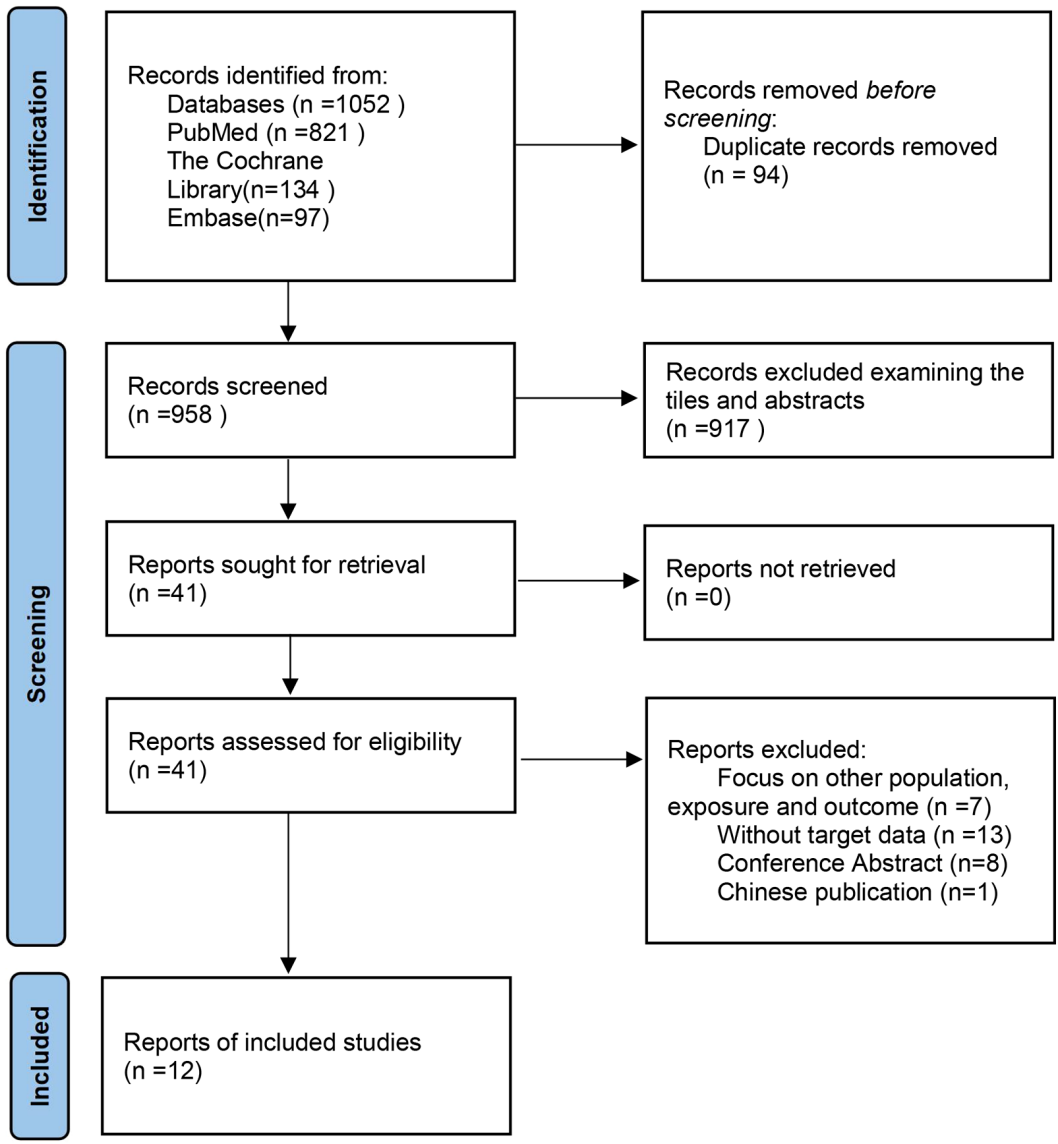
Flow chart of the study selection process.

We made the process of literature search and screening a flow chart (**Figure 1**). A total of 1,052 articles were initially retrieved. After reading the title and abstract of the article, 1,011 studies were excluded. We read the full text of the remaining 41 texts, while 28 of them were excluded for the following reasons: a) Focus on other population, exposure, and outcome (n =7) (10–16); b) Without target data (n =13) (17–29); c) Conference Abstract (n=8) (30–37); d) In Chinese (n=1) (38). All the detailed reasons for the excluded studies can be seen in **Supplemental Table 3**. Finally, 4 cohort studies (6, 39–41) and 8 cross-sectional studies (4, 5, 42–47) were included in the meta-analysis.

### Study characteristics and quality

The concrete characteristics of these included studies are shown in **Table 1**. Twelve studies with 28,788 NAFLD cases and 105,365 participants were included in this meta-analysis (4–6, 39–47). Overall, the sample sizes of the included studies ranged from 184 to 52,575, while the mean age ranged from 39.9 to 68.9 years. Published between 2017 and 2022, one of them was from America, and one was from Europe (France), while the others were all from Asia.

**Table 1:** Characteristics of included studies in this meta-analysis.

References (First Author, Year, Country/Region)	Source of participants	Participant Characteristics	NAFLD diagnosis	Study design	Case /N	Mean age (years), Male (%)	Mean BMI (kg/m²)	Categories of TyG	OR/HR (95% CIs)	Follow-Up Period	Adjustments
Zhang, 2017, China	WISCO	General population	Ultrasound	Cross-sectional study	1,630/ 6,809	48.4, 59.6	22.0	Continuous variable	2.10 (1.90-2.20)	NR	Age, gender, DBP, LDL-C, ALT, UA, WBC
Zhang, 2017, China^(2)^	WISCO	General population	Ultrasound	Cross-sectional study	4,349/ 10,761	49.5, 62.8	23.7	<8.00 8.10-8.40 8.50-8.90 >9.00	Ref 1.80 (1.50-2.10) 3.00 (2.50-3.50) 6.30 (5.30-7.50)	NR	Age, gender, MI, SBP, UA, WBC, ALT
Zheng, 2018, China	Zhenhai Lianhua Hospital	General population	Ultrasound	Prospective cohort	1,390/ 4,539	42.0, 66.0	22.2	Men: ≤8.09 8.10-8.40 8.41-8.76 ≥8.77 Women ≤7.85 7.86-8.15 8.16-8.50 ≥8.51 Continuous variable	Ref 1.46(1.21-1.77) 2.49(2.08-2.97) 3.95(3.34-4.68) Ref 1.46(1.21-1.77) 2.49(2.08-2.97) 3.95(3.34-4.68) 5.72 (4.65-7.03)	9 years	Age, gender, WC, BMI, SBP, DBP, TC, TG, HDL-C, LDL-C, Apo-A1, Apo-B, FPG, BUN, Cr, AST, ALT, γ-GGT, UA and eGFR
Kitae, 2019, Japan	Asahi University Hospital	General population	Ultrasound	Retrospective cohort	2,670/ 14,086	40.0, 48.5	21.2	<8.00 8.00-8.48 ≥8.48	Ref 1.42(1.23-1.64) 1.93(1.68-2.22)	NR	Age, BMI, alcohol consumption, exercise, smoking, ALT
Lee, 2019, Korea	Gangnam Severance Hospital Health Promotion Center	General population	Ultrasound	Cross-sectional study	2,069/ 4,986	52.6, 59.7	23.84	≤8.04 8.05-8.42 8.43-8.81 ≥8.82 Continuous variable	Ref 1.57 (1.26–1.94) 2.32 (1.87–2.88) 2.94 (2.32–3.72) 5.70 (4.67-6.95)	NR	Age, gender, BMI, SBP, TC, HDL-C, ALT, presence of hypertension
Huanan, 2020, China	Xinzheng, Henan Province	General population	Ultrasound	Retrospective cohort	5,660/ 46,693	68.9, 48.9	23.9	≤8.11 8.12-8.44 8.45-8.78 >8.79 Continuous variable	Ref 0.98 (0.90-1.07) 1.09 (1.00-1.19) 1.31 (1.23-1.46) 1.27 (1.20-1.33)	3.19 years	Age, gender, living alone, current smoking, exercise, waist-to-height ratio, SBP, DBP, ALT, AST, TB, TC, and diabetes
Choe, 2020, Korea	A university hospital in South Korea	Patients with CKD	Ultrasound	Cross-sectional study	140/ 819	64.7, 58.9	24.8	Continuous variable	4.90(3.05-7.89)	NR	NR
Khamseh, 2021, USA	NR	General population	Ultrasound	Cross-sectional study	96/ 184	44.7, 50.5	30.5	Continuous variable	2.55 (1.02-6.38)	NR	Age, gender, waist-to-hip ratio, SBP, DBP, serum cholesterol, ALT, AST, HOMA-IR, statin medication, smoking and physical activity.
Lin, 2021, Taiwan	southern Taiwan	General population	Ultrasound	Cross-sectional study	826/ 1,969	54.9, 38.8	24.9	Continuous Variable	Men 2.89(2.13-3.91) Women 4.49(3.39-5.96)	NR	Age, AST, ALT, TC, hemoglobin, eGFR and UA
Sheng, 2021, China	the NAGALA Study (Murakami Memorial Hospital)	General population	Ultrasound	Cross-sectional study	2,507/ 14,281	44.1, 51.9	22.02	Continuous variable	Men 1.79(1.63-1.95) Women 2.43 (2.06-2.86)	NR	Age, habit of exercise, GGT, TC, HDL-C, HbA1c, smoking status, drinking status and DBP
Riviere, 2022, France	COMET biobank	Patients with obesity	Ultrasound	Cohort study	159/ 238	43.0, 33.6	42.0	Continuous variable	2.00(1.10-3.64)	NR	Age, gender, ASAT, GGT and platelet
Kim, 2022, Korea	Kangbuk Samsung Health Study cohort	General population	Ultrasound	Prospective cohort	7,292/ 52,575	39.9, 53.9	22.23	Continuous variable	1.25 (1.16-1.34)	5.1 years	Age, gender, AST, ALT, HDL-C, SBP, daily alcohol consumption, current smoking, regular physical activity, hypertension, hypercholesterolemia, and creatinine.

NAFLD, nonalcoholic fatty liver disease; BMI, body mass index; TyG, triglyceride and glucose index; NR, not reported. WISCO, Wuhan Iron and Steel Company; DBP, diastolic blood pressure; LDL-C, low density lipoprotein cholesterol; ALT, alanine aminotransferase; UA, uric acid; WBC, white blood cell; SBP, systolic blood pressure; MS, metabolic syndrome; WC, Waist circumference; TC, total cholesterol; TG, triglyceride; FPG, fasting plasma glucose; BUN, blood urea nitrogen; HDL-C, high density lipoprotein cholesterol; AST, aspartate transferase; γ-GGT,γ-glutamyltransferase; eGFR, estimated glomerular filtration rate; TB, total bilirubin; HOMA-IR, homeostasis model assessment of insulin resistance; HbA1c, hemoglobin A1c; ASAT, aspartate aminotransferase; GGT, gamma-glutamyl transpeptidase.

Among these articles, 8 cross-sectional studies were assessed by the Joanna Briggs Institute critical appraisal checklist (**Supplemental Table 4**). One of them (42) did not consider confounding factors, and it is not clear whether three of them (4, 43, 45) identified subpopulations by objective criteria. The rest were cohort studies that were evaluated by NOS, all scored as high quality with an NOS of more than 6 stars (**Supplemental Table 5**).

### Dose−response analysis between TyG and NAFLD

**Figure 2:**
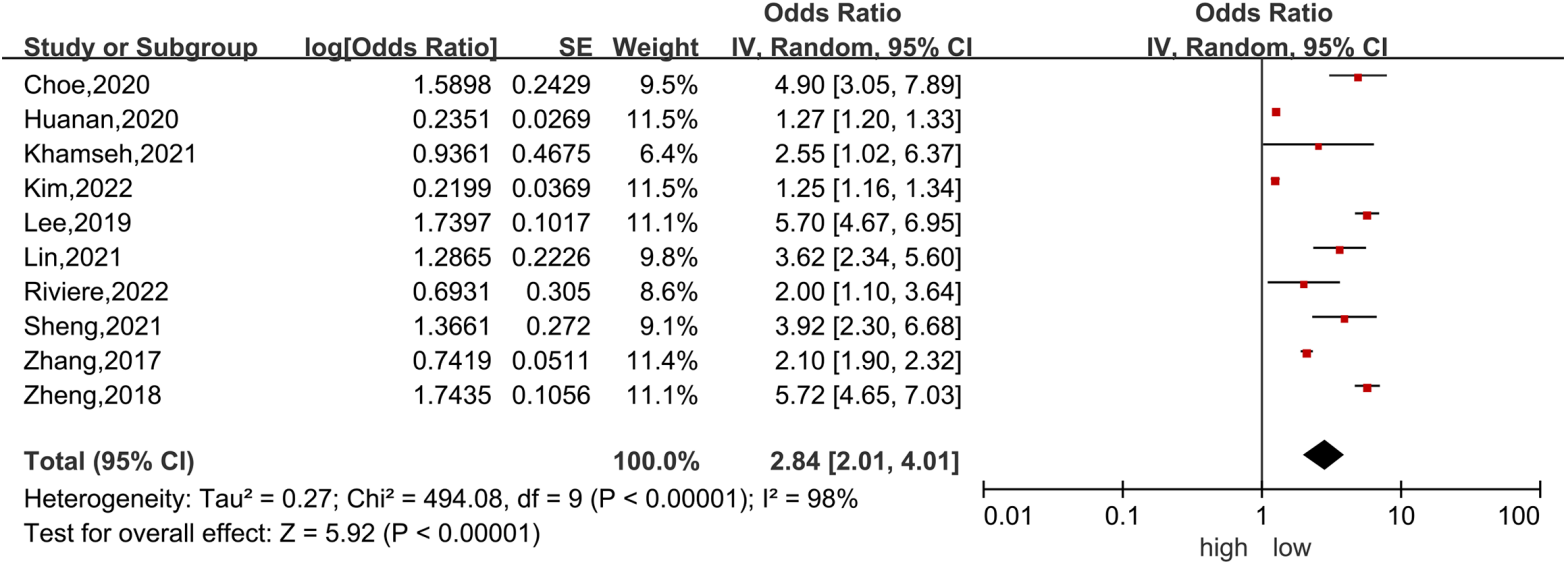
Forest plot of the association between each 1-unit increase in the triglyceride-glucose index and the risk of nonalcoholic fatty liver disease. The black midline indicates the line of no effect. The diamond indicates the pooled estimate. Gray boxes are relative to study size, and the black transverse lines indicate the 95% confidence interval around the effect size estimate.

**Figure 3:**
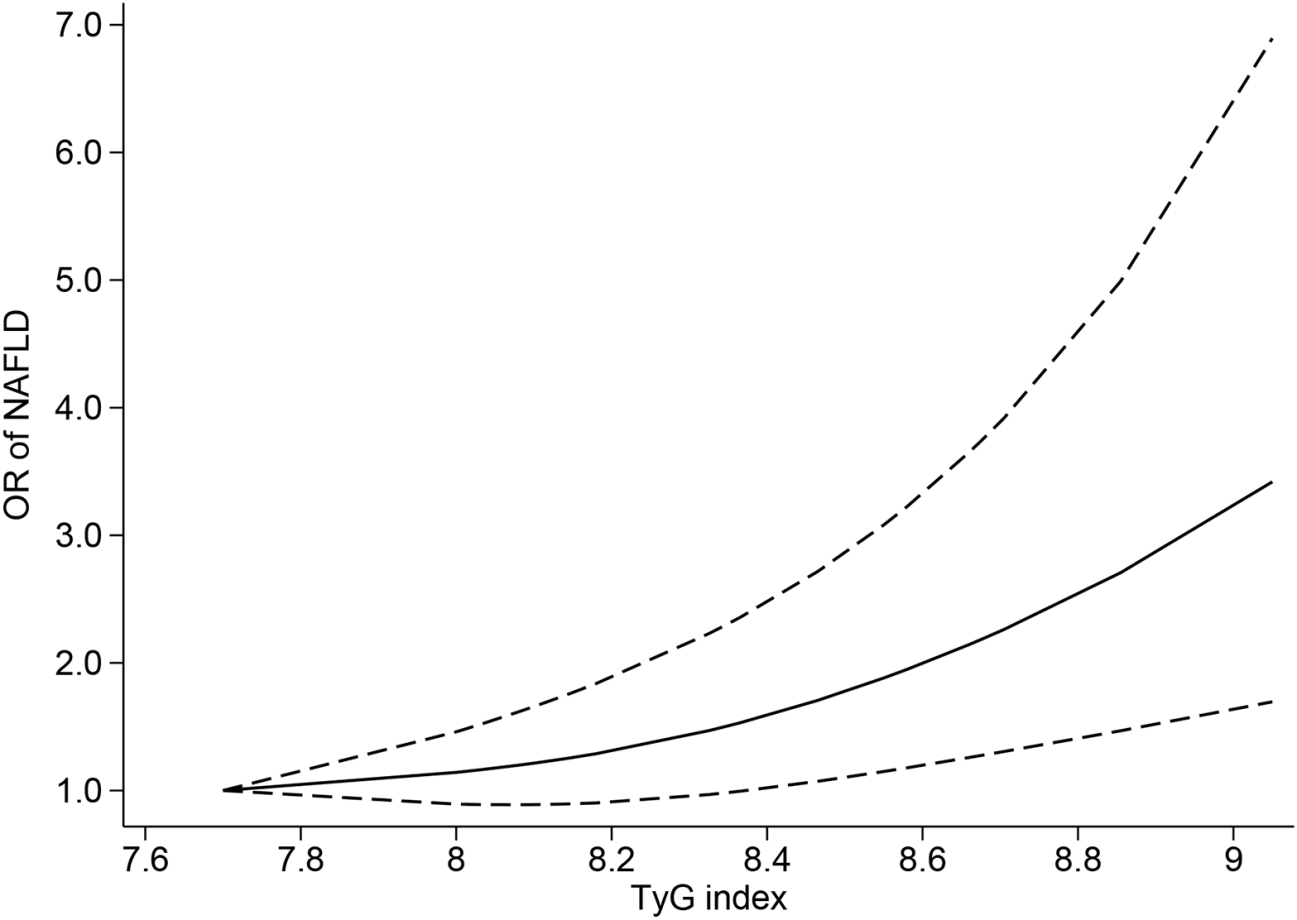
Triglyceride-glucose index and risk of nonalcoholic fatty liver disease, linear dose−response analysis. The solid line and the dashed lines represent the estimated relative risk and the 95% confidence interval, respectively.

Ten studies (4, 6, 39–43, 45–47) were included in the dose−response analysis of TyG and NAFLD. The summary OR for each 1-unit increase in TyG was 2.84 (95% CI, 2.01-4.01, I^2 =^ 98%, P<0.001; **Figure 2**), which suggested that the association between the TyG index and the risk of NAFLD was significant. A positive relationship is shown in **Figure 3** between TyG and the risk of NAFLD with evidence of linearity, Pnon-linearity =0.82, which was more than 0.05. **Supplementary Table 6** shows the estimated OR derived from the linear curve of dose−response analysis for the TyG index and NAFLD.

### Sensitivity analysis and publication bias

The combined results were consistent with the original results when any study was omitted from the sensitivity analysis (**Supplementary Figure 1**). Some indication of publication bias can be discovered when using Egger’s test (P=0.044) or by inspecting the asymmetrical funnel plot. However, there was no evidence of publication bias shown in Begg’s test (P=0.858) (**Supplementary Figure 2**). Then, the “trim-and-fill” method was used for the adjustment of publication bias in our analysis. After 3 iterations using the linear method, the data of the five virtual studies were entered; however, the results remained significant (OR=1.52 95% CI, 1.08-2.13).

### Subgroup analyses

Subgroup analyses according to age, study design, sample size, BMI and adjustment for confounders are shown in **Table 2**. Heterogeneity was not evident in the sex-specific groups, suggesting that sex may be a potential source of heterogeneity across the main results. The summary OR for women was 4.80 (95% CI, 3.90-5.90), which was higher than the pooled result for men of 2.97 (95% CI, 2.55-3.46), suggesting a stronger association between the TyG index and NAFLD in females (P<0.001). Additionally, in the subgroup analyses defined by adjustment for confounding factors, a great difference was shown in the group adjusted for BMI, diabetes, and exercise (P<0.001).

**Table 2:** Subgroup analysis of TyG and risk of NAFLD.

Items		Number of studies	ES (95%CI)	P	${\text{P}}_{*}^{\text{h}}$ (%)	P^#^
Result of primary analysis		10	2.84 [2.01, 4.01]	<0.001	98	–
Mean age	<60 years	8	2.99 [1.84, 4.86]	<0.001	98	0.78
	≥60 years	2	2.44 [0.65, 9.19]	0.19	97	–
Study design	Cohort	4	2.04 [1.32, 3.14]	0.001	98	0.09
	Cross-sectional	6	3.63 [2.19, 6.02]	<0.001	94	–
Sample size	<2000	4	3.28 [2.21, 4.86]	<0.001	48	0.49
	≥2000	6	2.68 [1.76, 4.07]	<0.001	99	–
Region	Europe	1	2.00 [1.10, 3.64]	0.02	–	0.55
	America	1	2.55 [1.02, 6.37]	0.05	–	–
	Asia	8	2.97 [2.04, 4.33]	<0.001	99	–
Source of participants	Medical Institutions	6	3.41 [1.53, 7.59]	0.003	99	0.29
	Community	4	2.09 [1.37, 3.19]	<0.001	97	–
BMI	<30	8	2.97 [2.04, 4.33]	<0.001	99	0.31
	≥30	2	2.15 [1.30, 3.55]	0.003	0	–
Gender	Men	2	2.97 [2.55, 3.46]	<0.001	0	<0.001
	Women	2	4.80 [3.90, 5.90]	<0.001	0	–
Adjustment for confounders						
Age	Yes	9	2.68 [1.87, 3.84]	<0.001	98	0.05
	No	1	4.90 [3.05, 7.89]	<0.001	–	–
Gender	Yes	7	2.45 [1.65, 3.65]	<0.001	99	0.04
	No	3	4.09 [3.11, 5.39]	<0.001	0	–
BMI	Yes	2	5.71 [4.94, 6.59]	<0.001	0	<0.001
	No	8	2.19 [1.70, 2.82]	<0.001	95	**-**
SBP	Yes	5	2.63 [1.57, 4.42]	<0.001	99	0.65
	No	5	3.06 [2.06, 4.54]	<0.001	81	–
ALT	Yes	7	2.65 [1.78, 3.94]	<0.001	99	0.42
	No	3	3.46 [2.08, 5.75]	<0.001	63	–
UA	Yes	3	3.50 [1.66, 7.36]	<0.001	97	0.46
	No	7	2.56 [1.75, 3.75]	<0.001	98	–
HOMA-IR	Yes	1	2.55 [1.02, 6.37]	0.05	–	0.82
	No	9	2.86 [2.00, 4.09]	<0.001	98	–
Hypertension	Yes	2	2.66 [0.60, 11.78]	<0.001	99	0.91
	No	8	2.91 [1.88, 4.50]	<0.001	98	–
Diabetes	Yes	1	1.27 [1.20, 1.33]	<0.001	–	<0.001
	No	9	3.16 [1.99, 5.01]	<0.001	98	–
Exercise	Yes	4	1.42 [1.19, 1.69]	<0.001	85	<0.001
	No	6	3.71 [2.24, 6.14]	<0.001	96	–

NAFLD, nonalcoholic fatty liver disease; TyG, triglyceride and glucose index; BMI, body mass index; SBP, systolic blood pressure; ALT, alanine aminotransferase; UA, uric acid; HOMA-IR, homeostasis model assessment of insulin resistance; *P for within-group heterogeneity, #P for subgroup difference

## Discussion

### Major findings

Our study found that the positive association between TyG and the risk of NAFLD in a linear model was strong, and for additional units of TyG, the risk of NAFLD increased by 2.84 times. To the best of our knowledge, this is the first time that the dose−response relationship between the TyG index and the risk of NAFLD has been presented. Our result is consistent with a previous meta-analysis of large observational studies (48). In addition, we evaluated the dose−response relationship between the TyG index and NAFLD for the first time, identifying a specific value for the increased risk of NAFLD that was caused by the per unit increase in the TyG index. Therefore, our study can provide new ideas for the detection and prevention of NAFLD and can also determine specific cut-off values, which is of great significance in clinical application.

With respect to sex in the subgroup analysis, we found that women had a 1.6 times higher risk of NAFLD than men for each additional unit of TyG. This finding caught our attention because women have more peripheral and subcutaneous fat than visceral and hepatic adipose tissue, and combined with the protective effect of estrogen on NAFLD, women may have a lower risk of NAFLD than men (49). The contradiction may come from the mean age of the populations included in this subgroup, which was more than 44 years old in both included studies. Most women at this age are going through menopause, accompanied by decreasing estrogen levels (50). Studies have shown that postmenopausal women have a higher prevalence of NAFLD than men due to the higher possibility of weight gain, fat redistribution, and dyslipidemia, all of which can contribute to an increased risk of NAFLD (51). Moreover, our results highlighted the changes in NAFLD when increasing the same amount of TyG in different sexes, which may be related to sex differences in increased carbohydrate and lipid metabolism, as well as menopausal changes in body fat morphology and increased susceptibility to metabolic complications (52). However, considering the limited number of sex subgroups (N=2), further research is needed to confirm the sex difference in the TyG index with NAFLD.

In the subgroup for confounding factors, there will be a great difference across adjustments stratified by BMI, sedentary lifestyle, and diabetes. These results were not surprising. BMI is an important indicator to assess obesity, and obesity is recognized to be closely associated with NAFLD (53). Moreover, a sedentary lifestyle (54) and diabetes (55) are also hazards for NAFLD. Hence, BMI, sedentary lifestyle and diabetes may be modifiers of the relationship of the TyG index with NAFLD.

### Potential mechanism

**Figure 4:**
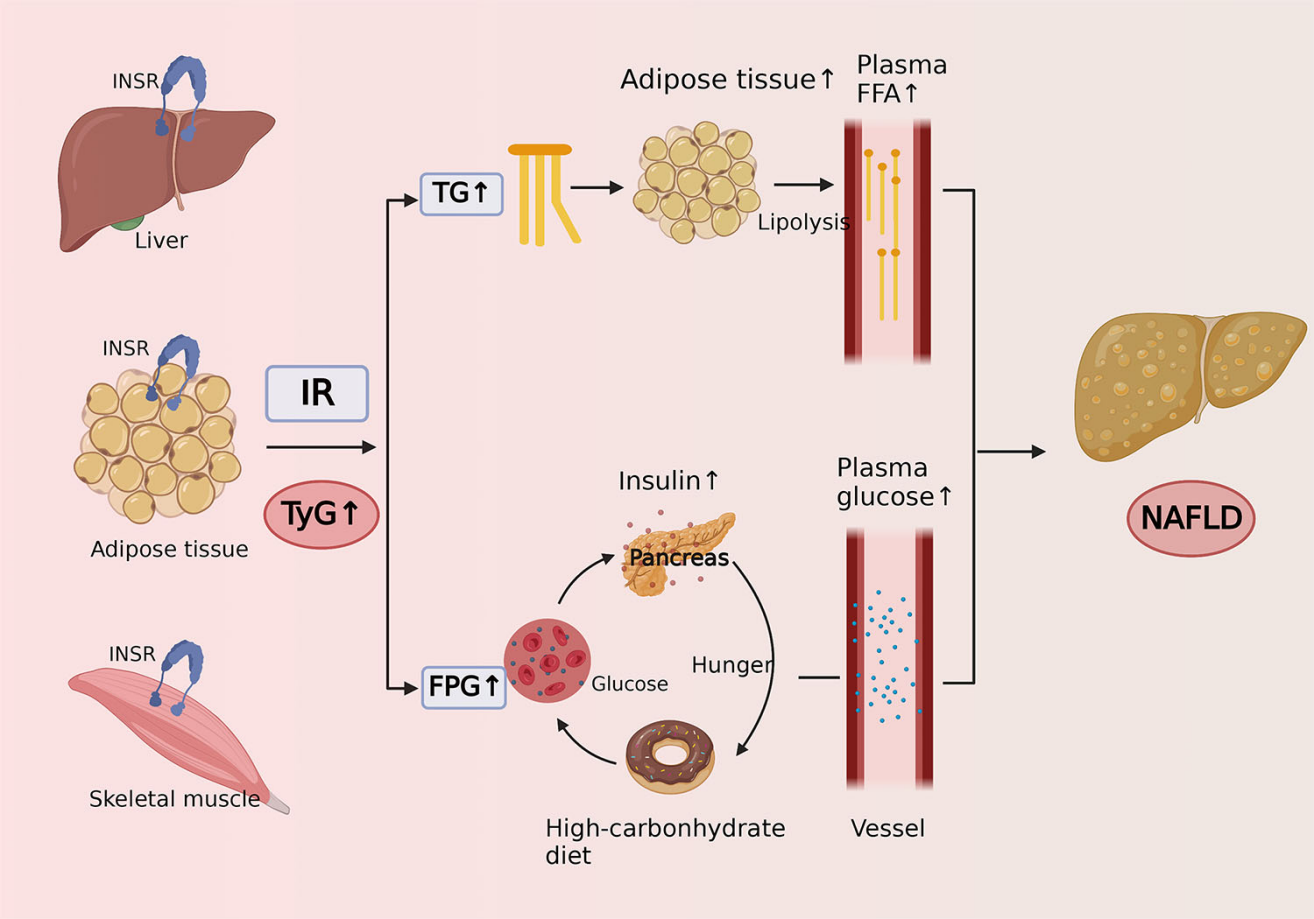
Cartoon describing the potential mechanism of the association between triglyceride-glucose index and risk of nonalcoholic fatty liver disease.

The TyG index is calculated by fasting triglycerides and fasting plasma glucose, and it is highly sensitive and specific for the identification of IR and has been widely used as a reliable alternative indicator for IR in recent years (56). The potential pathophysiological mechanisms of the association between the TyG index and the risk of NAFLD are as follows (**Figure 4**). It is widely recognized that NAFLD is closely related to IR (57), which mainly occurs in the liver, adipose, and muscle tissue. The excess blood glucose caused by IR will be converted into fat and increase triglycerides accordingly, which can promote lipolysis to raise the level of free fatty acids (58). Excess fatty acids are transported through the blood to the liver and further synthesized into fat, causing extra lipid deposition in the liver and contributing to NAFLD. In addition, due to the decreased sensitivity of insulin in patients with IR, circulating glucose will remain at a high level for a long time, promoting the secretion of insulin and stimulating hunger (59). As a result, the patients will become more eager to strive for a high-carbohydrate diet, thus forming a vicious cycle.

### Clinical implications

Both NAFLD and a high level of IR are associated with various diseases, such as liver cancer and many extraliver cancers, so the early detection of NAFLD may possibly alleviate or cure the potentially poor prognosis of this disease (60). The international diagnostic gold standard for NAFLD is liver biopsy (61), but it is invasive, complicated, and takes a long time to obtain results. Ultrasound, the technique commonly used in the clinical diagnosis of NAFLD, is mainly based on the grayscale to judge the lesion and its degree with the naked eye, which has shortcomings such as strong operation dependence and poor objectivity (62). Therefore, there is an urgent need for a noninvasive, accurate, and easy-to-judge method to diagnose NAFLD. The TyG index is a rapid, feasible, and applicable daily biomarker that can be obtained in routine medical examinations, and regular blood tests can effectively monitor the TyG index without extra cost. A study by Sheng et al (46) analysed the diagnostic performance of the TyG index for NAFLD, resulting in an area under the curve (AUC) of 0.98 (95% CI, 0.97-0.99). Moreover, Kim et al (27) and Zheng et al (6) also obtained high AUC values of 0.77 (95% CI, 0.76-0.78) and 0.76 (95% CI, 0.74-0.77), respectively. As a result, the TyG index may be an applicable tool to diagnose subjects with NAFLD noninvasively, and it may also serve as a good predictor for the risk of NAFLD. Some other studies have shown that TyG also has an association with the progression of NAFLD (15), and the role of TyG in the severity of NAFLD diagnosis should be verified in the future.

### Limitation

In general, most of our included studies were cross-sectional, which cannot prove a causal relationship (63). Although we only included the study of multivariate analysis, the remaining confounding factors will still affect our results. More studies with prospective designs are needed to confirm their association. Second, in patients with hyperglycemia, hyperlipidemia, or diabetes, the TyG index would be affected by these medications. However, due to the limitation of the number of included studies, the effect cannot be eliminated. In addition, due to the limitation of the number of studies, we cannot make a restriction or adjustment for factors such as the diet and lifestyle of the included population, which may have a profound impact on the TyG index. Moreover, only two of the included studies were conducted in Europe and America, while the majority were from Asia. As a result, more studies are needed to study the regional differences in the relationship between the TyG index and NAFLD.

## Conclusion

Our dose−response analysis suggested that the TyG index may be a new risk factor for NAFLD independent of traditional risk factors. However, the association may be affected by some confounding factors due to the limitations of our study, so more prospective studies are necessary to confirm this result.

## Data availability statement

The original contributions presented in the study are included in the article/**Supplementary Material**. Further inquiries can be directed to the corresponding authors.

## Author contributions

J-L, FW-L and P-Y participated in the whole project and were responsible for revising the draft. QL and JW-C X-L conducted the study selection, data extraction, statistical analysis, and interpretation of the data. Q-L and X-L wrote the first draft of the manuscript. All authors participated in the interpretation of the results and in revising the final version of the manuscript.
