# Supplementary material for: The triglyceride and glucose index and risk of nonalcoholic fatty liver disease: A dose–response meta-analysis
Source: Front Endocrinol (Lausanne). 2023 Jan 19;13:1043169. doi: 10.3389/fendo.2022.1043169 (PMC9892833; doi:10.3389/fendo.2022.1043169)
Supplement: Supplementary file 1 [file DataSheet_1.docx]

**The triglyceride and glucose index and risk of nonalcoholic fatty liver disease: a dose response meta-analysis**

**Supplementary Table 1. PRISMA Checklist.**

| **Section/topic** | **#** | **Checklist item** | **Reported on page #** |
| --- | --- | --- | --- |
| **TITLE** | | |  |
| Title | 1 | Identify the report as a systematic review, meta-analysis, or both. | 1 |
| **ABSTRACT** | | |  |
| Structured summary | 2 | Provide a structured summary including, as applicable: background; objectives; data sources; study eligibility criteria, participants, and interventions; study appraisal and synthesis methods; results; limitations; conclusions and implications of key findings; systematic review registration number. | 3 |
| **INTRODUCTION** | | |  |
| Rationale | 3 | Describe the rationale for the review in the context of what is already known. | 4-5 |
| Objectives | 4 | Provide an explicit statement of questions being addressed with reference to participants, interventions, comparisons, outcomes, and study design (PICOS). | 5 |
| **METHODS** | | |  |
| Protocol and registration | 5 | Indicate if a review protocol exists, if and where it can be accessed (e.g., Web address), and, if available, provide registration information including registration number. | 4-5 |
| Eligibility criteria | 6 | Specify study characteristics (e.g., PICOS, length of follow-up) and report characteristics (e.g., years considered, language, publication status) used as criteria for eligibility, giving rationale. | 5 |
| Information sources | 7 | Describe all information sources (e.g., databases with dates of coverage, contact with study authors to identify additional studies) in the search and date last searched. | 5 |
| Search | 8 | Present full electronic search strategy for at least one database, including any limits used, such that it could be repeated. | 5 |
| Study selection | 9 | State the process for selecting studies (i.e., screening, eligibility, included in systematic review, and, if applicable, included in the meta-analysis). | 5 |
| Data collection process | 10 | Describe method of data extraction from reports (e.g., piloted forms, independently, in duplicate) and any processes for obtaining and confirming data from investigators. | 6 |
| Data items | 11 | List and define all variables for which data were sought (e.g., PICOS, funding sources) and any assumptions and simplifications made. | 6 |
| Risk of bias in individual studies | 12 | Describe methods used for assessing risk of bias of individual studies (including specification of whether this was done at the study or outcome level), and how this information is to be used in any data synthesis. | 7 |
| Summary measures | 13 | State the principal summary measures (e.g., risk ratio, difference in means). | 6 |
| Synthesis of results | 14 | Describe the methods of handling data and combining results of studies, if done, including measures of consistency (e.g., I^2^) for each meta-analysis. | 7 |
| Risk of bias across studies | 15 | Specify any assessment of risk of bias that may affect the cumulative evidence (e.g., publication bias, selective reporting within studies). | 7 |
| Additional analyses | 16 | Describe methods of additional analyses (e.g., sensitivity or subgroup analyses, meta-regression), if done, indicating which were pre-specified. | 7 |
| **RESULTS** | | |  |
| Study selection | 17 | Give numbers of studies screened, assessed for eligibility, and included in the review, with reasons for exclusions at each stage, ideally with a flow diagram. | 7 |
| Study characteristics | 18 | For each study, present characteristics for which data were extracted (e.g., study size, PICOS, follow-up period) and provide the citations. | 7-8 |
| Risk of bias within studies | 19 | Present data on risk of bias of each study and, if available, any outcome level assessment (see item 12). | 9 |
| Results of individual studies | 20 | For all outcomes considered (benefits or harms), present, for each study: (a) simple summary data for each intervention group (b) effect estimates and confidence intervals, ideally with a forest plot. | 8 |
| Synthesis of results | 21 | Present results of each meta-analysis done, including confidence intervals and measures of consistency. | 8 |
| Risk of bias across studies | 22 | Present results of any assessment of risk of bias across studies (see Item 15). | 9 |
| Additional analysis | 23 | Give results of additional analyses, if done (e.g., sensitivity or subgroup analyses, meta-regression [see Item 16]). | 9 |
| **DISCUSSION** | | |  |
| Summary of evidence | 24 | Summarize the main findings including the strength of evidence for each main outcome; consider their relevance to key groups (e.g., healthcare providers, users, and policy makers). | 9-12 |
| Limitations | 25 | Discuss limitations at study and outcome level (e.g., risk of bias), and at review-level (e.g., incomplete retrieval of identified research, reporting bias). | 13 |
| Conclusions | 26 | Provide a general interpretation of the results in the context of other evidence, and implications for future research. | 13 |
| **FUNDING** | | |  |
| Funding | 27 | Describe sources of funding for the systematic review and other support (e.g., supply of data); role of funders for the systematic review. | 14 |

*From:*  Moher D, Liberati A, Tetzlaff J, Altman DG, The PRISMA Group (2009). Preferred Reporting Items for Systematic Reviews and Meta-Analyses: The PRISMA Statement. PLoS Med 6(7): e1000097. doi:10.1371/journal.pmed1000097

For more information, visit: **www.prisma-statement.org**.

**Supplementary Table 2. Search strategy**

**PubMed database**

| **Search** | **Query** |
| --- | --- |
| **#1** | **((((((((((((("Non-alcoholic Fatty Liver Disease"[Mesh]) OR (Non alcoholic Fatty Liver Disease)) OR (NAFLD)) OR (Nonalcoholic Fatty Liver Disease)) OR (Fatty Liver, Nonalcoholic)) OR (Fatty Livers, Nonalcoholic)) OR (Liver, Nonalcoholic Fatty)) OR (Livers, Nonalcoholic Fatty)) OR (Nonalcoholic Fatty Liver)) OR (Nonalcoholic Fatty Livers)) OR (Nonalcoholic Steatohepatitis)) OR (Nonalcoholic Steatohepatitides)) OR (Steatohepatitides, Nonalcoholic)) OR (Steatohepatitis, Nonalcoholic)** |
| **#2** | **((((TyG index) OR (triglyceride glucose index)) OR (triacylglycerol glucose index)** |
| **#3** | **#1 AND #3** |

**Embase database**

| **Search** | **Query** |
| --- | --- |
| **#1** | **'nonalcoholic fatty liver’/exp** |
| **#2** | **'non alcoholic fatty liver disease' OR 'nafld' OR 'nonalcoholic fatty liver disease' OR 'fatty liver, nonalcoholic' OR 'fatty livers, nonalcoholic' OR 'liver, nonalcoholic fatty' OR 'ivers, nonalcoholic fatty' OR 'nonalcoholic fatty liver' OR 'nonalcoholic fatty livers' OR 'nonalcoholic steatohepatitis' OR 'nonalcoholic steatohepatitides' OR 'steatohepatitides, nonalcoholic' OR 'steatohepatitis, nonalcoholic'** |
| **#3** | **#1 OR #2** |
| **#4** | **'tyg index' OR 'triglyceride glucose index' OR 'triacylglycerol glucose index'** |
| **#5** | **#3 AND #4** |

**Cochrane library**

| **Search** | **Query** |
| --- | --- |
| **#1** | **MeSH descriptor: [Non-alcoholic Fatty Liver Disease] explode all trees** |
| **#2** | **(“non-alcoholic fatty liver disease”): ti,ab,kw** |
| **#3** | **#1 OR #2** |
| **#4** | **(“triglyceride glucose index”): ti,ab,kw** |
| **#5** | **#3 AND #4** |

**Supplementary Table 3. Studies excluded (n=29) with reasons**

| **Studies excluded** | **Reasons (according to PICOS)** |
| --- | --- |
| Song, 2022[1] | Focus on other population: Youths |
| Son, 2022[2] | Without target data set: Not providing OR, HR or RR |
| Li,2022[3] | Without target data set: Not available for dose-response analysis |
| Katalin, 2022[4] | Conference Abstract |
| Amzolini, 2022[5] | Without target data set: Not providing OR, HR or RR |
| Ye, 2021[6] | Without target data set: Not providing OR, HR or RR |
| Wang, R, 2021[7] | Focus on other exposure: triglyceride glucose-body mass index |
| Wang, J, 2021[8] | Without target data set: Not available for dose-response analysis |
| Smiderle, 2021[9] | Without target data set: Not available for dose-response analysis |
| Simental, 2021[10] | Focus on other population: Children |
| Shi, 2021[11] | Focus on other population: Children |
| Liu, Y, 2021[12] | Use other language besides English: in Chinese |
| Liu, J, 2021[13] | Focus on other outcome: progression and improvement of NAFLD |
| Kim, 2021[14] | Without target data set: Not available for dose-response analysis |
| Amzolini, 2021[15] | Without target data set: Not providing OR, HR or RR |
| Tutunchi, 2021[16] | Focus on other population: people with NAFLD |
| Zhu, 2020[17] | Without target data set: Not available for dose-response analysis |
| Runderawala,2020[18] | Conference Abstract |
| Li, 2020[19] | Focus on other exposure: triglyceride glucose-body mass index |
| Guo, 2020[20] | Without target data set: Not available for dose-response analysis |
| Cen, 2020[21] | Without target data set: Not providing OR, HR or RR |
| Alasadi, 2020[22] | Without target data set: Not providing OR, HR or RR |
| Lim, 2019[23] | Conference Abstract |
| Kim,2019[24] | Conference Abstract |
| Kim, 2018[25] | Conference Abstract |
| Inavolu, P, 2018[26] | Conference Abstract |
| Inavolu, P, 2018^(2)^[27] | Conference Abstract |
| Kim, 2017[28] | Conference Abstract |
| Simental, 2016[29] | Without target data set: Not providing OR, HR or RR |

[1] K. Song, G. Park, H.S. Lee, M. Lee, H.I. Lee, H.S. Choi, J. Suh, A. Kwon, H.S. Kim, and H.W. Chae, Comparison of the Triglyceride Glucose Index and Modified Triglyceride Glucose Indices to Predict Nonalcoholic Fatty Liver Disease in Youths. The Journal of pediatrics 242 (2022) 79-85.e1.

[2] D.H. Son, H.S. Lee, Y.J. Lee, J.H. Lee, and J.H. Han, Comparison of triglyceride-glucose index and HOMA-IR for predicting prevalence and incidence of metabolic syndrome. Nutrition, Metabolism and Cardiovascular Diseases 32 (2022) 596-604.

[3] N. Li, H. Tan, A. Xie, C. Li, X. Fu, W. Xang, A. Kirim, and X. Huang, Value of the triglyceride glucose index combined with body mass index in identifying non-alcoholic fatty liver disease in patients with type 2 diabetes. BMC endocrine disorders 22 (2022) 101.

[4] C. Katalin, and B. Ágnes, TyG index correlation with surrogate measures of visceral obesity, insulin resistance and non-alcoholic fatty liver disease. Metabolism: clinical and experimental 128 (2022).

[5] A.M. Amzolini, M.C. Forțofoiu, A.B. Alhija, I.M. Vladu, D. Clenciu, A. Mitrea, M. Forțofoiu, D. Matei, M. Diaconu, M.S. Tudor, and E.S. Micu, Triglyceride and Glucose Index as a Screening Tool for Nonalcoholic Liver Disease in Patients with Metabolic Syndrome. Journal of clinical medicine 11 (2022).

[6] X. Ye, J. Li, H. Wang, and J. Wu, Pentraxin 3 and the TyG Index as Two Novel Markers to Diagnose NAFLD in Children. Disease markers 2021 (2021) 8833287.

[7] R. Wang, L. Dai, Y. Zhong, and G. Xie, Usefulness of the triglyceride glucose-body mass index in evaluating nonalcoholic fatty liver disease: insights from a general population. Lipids in health and disease 20 (2021) 77.

[8] J. Wang, Z. Su, Y. Feng, R. Xi, J. Liu, and P. Wang, Comparison of several blood lipid-related indexes in the screening of non-alcoholic fatty liver disease in women: a cross-sectional study in the Pearl River Delta region of southern China. BMC gastroenterology 21 (2021) 482.

[9] C.A. Smiderle, G.P. Coral, D.E.C. LA, A.A. Mattos, A.Z. Mattos, and C.V. Tovo, PERFORMACE OF TRIGLYCERIDE-GLUCOSE INDEX ON DIAGNOSIS AND STAGING OF NAFLD IN OBESE PATIENTS. Arquivos de gastroenterologia 58 (2021) 139-144.

[10] L.E. Simental-Mendía, C.J. Ortega-Pacheco, E. García-Guerrero, M.A. Sicsik-Aragón, F. Guerrero-Romero, and G. Martínez-Aguilar, The triglycerides and glucose index is strongly associated with hepatic steatosis in children with overweight or obesity. European journal of pediatrics 180 (2021) 1755-1760.

[11] M. Shi, P. Liu, J. Li, Y. Su, X. Zhou, C. Wu, X. Chen, and C. Zheng, The performance of noninvasive indexes of adults in identification of nonalcoholic fatty liver disease in children. Journal of diabetes 13 (2021) 744-753.

[12] Y.T. Liu, W. Wang, J. Tong, and B.Y. Wang, [Relationship between triglyceride-glucose index and non-alcoholic fatty liver disease]. Zhonghua gan zang bing za zhi = Zhonghua ganzangbing zazhi = Chinese journal of hepatology 29 (2021) 451-455.

[13] J. Liu, L. Guan, M. Zhao, Q. Li, A. Song, L. Gao, H. Lin, and J. Zhao, Association Between the Triglyceride-Glucose Index and Outcomes of Nonalcoholic Fatty Liver Disease: A Large-Scale Health Management Cohort Study. Diabetes, metabolic syndrome and obesity : targets and therapy 14 (2021) 2829-2839.

[14] H.S. Kim, Y.K. Cho, E.H. Kim, M.J. Lee, C.H. Jung, J.Y. Park, H.K. Kim, and W.J. Lee, Triglyceride Glucose-Waist Circumference Is Superior to the Homeostasis Model Assessment of Insulin Resistance in Identifying Nonalcoholic Fatty Liver Disease in Healthy Subjects. Journal of clinical medicine 11 (2021).

[15] A.M. Amzolini, M.C. Forţofoiu, A. Barău Abu-Alhija, I.M. Vladu, D. Clenciu, A. Mitrea, M. Forţofoiu, D. Matei, V. Enăchescu, O.I. Predescu, and E.S. Micu, Triglyceride and glucose index: a useful tool for non-alcoholic liver disease assessed by liver biopsy in patients with metabolic syndrome? Romanian journal of morphology and embryology = Revue roumaine de morphologie et embryologie 62 (2021) 475-480.

[16] H. Tutunchi, F. Naeini, M. Mobasseri, and A. Ostadrahimi, Triglyceride glucose (TyG) index and the progression of liver fibrosis: A cross-sectional study. Clinical nutrition ESPEN 44 (2021) 483-487.

[17] J. Zhu, D. Xu, R. Yang, M. Liu, and Y. Liu, The triglyceride glucose index (TyG) and CDKAL1 gene rs10946398 SNP are associated with NAFLD in Chinese adults. Minerva endocrinologica (2020).

[18] H. Runderawala, and N. Desai, The triglyceride and glucose index (TyG) as screening biomarker to identify non-alcoholic fatty liver disease. Indian Journal of Gastroenterology 39 (2020) S48-S49.

[19] Y. Li, R. Zheng, J. Li, S. Feng, L. Wang, and Z. Huang, Association between triglyceride glucose-body mass index and non-alcoholic fatty liver disease in the non-obese Chinese population with normal blood lipid levels: a secondary analysis based on a prospective cohort study. Lipids in health and disease 19 (2020) 229.

[20] W. Guo, J. Lu, P. Qin, X. Li, W. Zhu, J. Wu, N. Xu, and Q. Zhang, The triglyceride-glucose index is associated with the severity of hepatic steatosis and the presence of liver fibrosis in non-alcoholic fatty liver disease: a cross-sectional study in Chinese adults. Lipids in health and disease 19 (2020) 218.

[21] C. Cen, W. Wang, S. Yu, X. Tang, J. Liu, Y. Liu, L. Zhou, J. Yu, and S. Zheng, Development and validation of a clinical and laboratory-based nomogram to predict nonalcoholic fatty liver disease. Hepatology international 14 (2020) 808-816.

[22] A. Alasadi, H.H. Humaish, and H. Al-Hraishawi, Evaluation the predictors of non-alcoholic fatty liver disease (NAFLD) in type 2 diabetes mellitus (T2DM) patients. Systematic Reviews in Pharmacy 11 (2020) 421-430.

[23] J. Lim, Validation of fatty liver index in a healthy Korean population and its comparison with triglyceride glucose index and its related parameters. Clinica Chimica Acta 493 (2019) S374.

[24] K.S. Kim, Y.C. Hwang, H.Y. Ahn, S.W. Park, and C.Y. Park, Triglyceride and glucose (TYG) index is an effective predictor of nonalcoholic fatty liver disease/nonalcoholic steatohepatitis. Diabetes 68 (2019).

[25] M.K. Kim, J.H. Kim, K. Park, S.B. Lee, J.S. Nam, S. Kang, J.S. Park, C.W. Ahn, and Y.S. Kim, Relationship between the triglyceride glucose index and the presence and fibrosis of nonalcoholic fatty liver disease in Korean adults. Diabetes 67 (2018) A161.

[26] P. Inavolu, N. Singla, K. Nunsavata, and R.K. Bhashyakarla, Triglyceride and glucose index (TYG) index as an screening biomarker to identify nonalcoholic fatty liver disease. Journal of Clinical and Experimental Hepatology 8 (2018) S41.

[27] P. Inavolu, Screening of non-alcoholic fatty liver disease using new screening biomarker triglyceride and glucose index (Tyg) index. Indian Journal of Gastroenterology 37 (2018) A74.

[28] H.Y. Kim, D.R. Ryu, H.A. Lee, T.H. Kim, K. Yoo, J.S. Kim, and J.K. Lee, Noninvasive indices for the prediction of presence and severity of nonalcoholic fatty liver disease in patients with chronic kidney disease. Hepatology (Baltimore, Md.) 66 (2017) 1165A-1166A.

[29] L.E. Simental-Mendía, E. Simental-Mendía, H. Rodríguez-Hernández, M. Rodríguez-Morán, and F. Guerrero-Romero, The product of triglycerides and glucose as biomarker for screening simple steatosis and NASH in asymptomatic women. Annals of hepatology 15 (2016) 715-20.

**Supplementary Table 4. Joanna Briggs Institute critical appraisal checklist applied for included studies.**

| Study | Sample was representative? | Participants appropriately recruited? | Sample size was adequate? | Study subjects and the setting described | Data analysis conducted | Objective, standard criteria, reliably used? | Appropriate statistical analysis used? | Confounding factors/ subgroups/ differences identified and accounted? | Subpopulations identified using objective criteria |
| --- | --- | --- | --- | --- | --- | --- | --- | --- | --- |
| **Zhang,2017** | Yes | Yes | Yes | Yes | Yes | Yes | Yes | Yes | Yes |
| **Zhang,2017^(2)^** | Yes | Yes | Yes | Yes | Yes | Yes | Yes | Yes | Yes |
| **Lee, 2019** | Yes | Yes | Yes | Yes | Yes | Yes | Yes | Yes | Unclear |
| **Choe, 2020** | Yes | Yes | Yes | Yes | Yes | Yes | Yes | No | Yes |
| **Khamseh,2021** | Yes | Yes | Yes | Yes | Yes | Yes | Yes | Yes | Unclear |
| **Lin,2021** | Yes | Yes | Yes | Yes | Yes | Yes | Yes | Yes | Unclear |
| **Sheng,2021** | Yes | Yes | Yes | Yes | Yes | Yes | Yes | Yes | Yes |

**Supplementary Table 5. Quality assessment of the included studies by Newcastle–Ottawa scale.**

| Study | Selection | | | | Comparability | Outcome | | | Total |
| --- | --- | --- | --- | --- | --- | --- | --- | --- | --- |
|  | Exposed cohort | Nonexposed cohort | Ascertainment of exposure | Outcome of interest |  | Assessment of outcome | Length of follow-up | Adequacy of follow-up |  |
| **Zheng,2018** | * | * | * | * | * | * | * | * | 8 |
| **Kitae, 2019** | * | * | * | * | ** | * | * | * | 9 |
| **Huanan,2020** | * | * | * | * | * | * | * | * | 8 |
| **Riviere,2022** |  | * | * | * | * | * |  | * | 6 |
| **Kim,2022** | * | * | * | * | * | * | * | * | 8 |

Comparability: Obesity = *, Other control factors = *; Adequacy of follow-up: Follow-up rate≥80% = *.

**Supplementary Table 6. Odds ratio from the linear dose-response analysis**

| **TyG index**  **Outcome** | Risk of NAFLD |
| --- | --- |
|  | OR (95%CI) |
| 7.70 | (1.00-1.00) |
| 8.00 | (0.89-1.46) |
| 8.01 | (0.89-1.48) |
| 8.03 | (0.89-1.52) |
| 8.08 | (0.89-1.62) |
| 8.10 | (0.89-1.66) |
| 8.14 | (0.89-1.75) |
| 8.18 | (0.90-1.84) |
| 8.32 | (0.97-2.23) |
| 8.35 | (0.98-2.29) |
| 8.36 | (0.99-2.36) |
| 8.47 | (1.07-2.72) |
| 8.55 | (1.15-3.08) |
| 8.58 | (1.18-3.22) |
| 8.66 | (1.26-3.65) |
| 8.68 | (1.27-3.74) |
| 8.70 | (1.31-3.92) |
| 8.86 | (1.47-4.99) |
| 9.05 | (1.70-6.89) |
| P_non-linearity_ | 0.8204 |

**
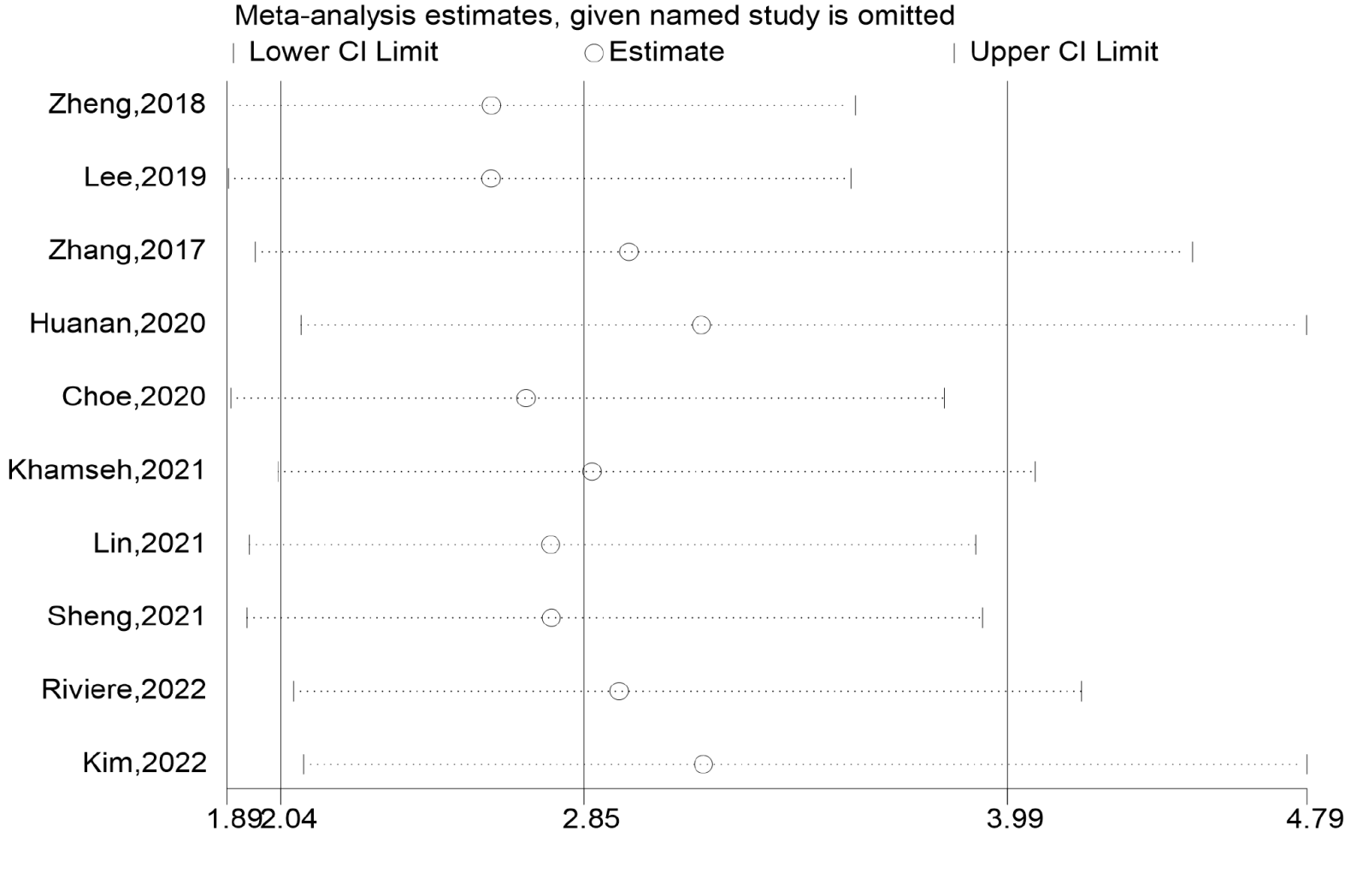
**

**Supplementary Figure 1. Sensitivity analysis of included studies that reported the association between TyG and the risk of NAFLD**

**
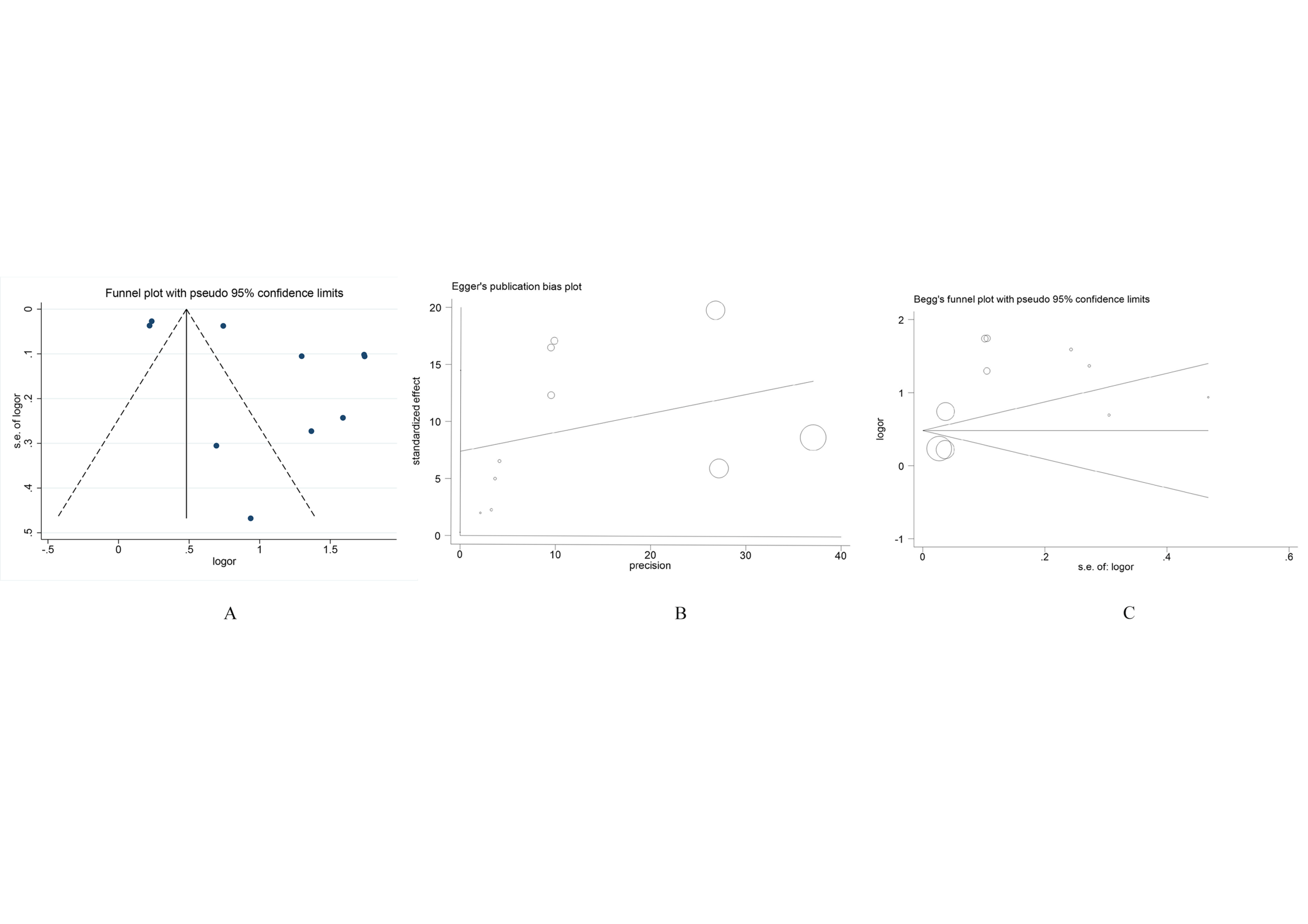
**

**Supplementary Figure 2. Publication bias detected by funnel plot, Egger’s test and Begg’s test for the association between TyG and the risk of NAFLD.**

A: Funnel plot; B: Egger’s test; C: begg’s test
